# Cryo-EM structure of the ASIC1a–mambalgin-1 complex reveals that the peptide toxin mambalgin-1 inhibits acid-sensing ion channels through an unusual allosteric effect

**DOI:** 10.1038/s41421-018-0026-1

**Published:** 2018-06-05

**Authors:** Demeng Sun, You Yu, Xiaobin Xue, Man Pan, Ming Wen, Siyu Li, Qian Qu, Xiaorun Li, Longhua Zhang, Xueming Li, Lei Liu, Maojun Yang, Changlin Tian

**Affiliations:** 1 School of Life Sciences, University of Science and Technology of China, Hefei, 230026 China; 2 Ministry of Education Key Laboratory of Protein Science, Beijing Advanced Innovation Center for Structural Biology, Tsinghua-Peking Joint Center for Life Sciences, School of Life Sciences, Tsinghua University, Beijing, 100084 China; 3 Ministry of Education Key Laboratory of Bioorganic Phosphorus Chemistry and Chemical Biology, Department of Chemistry, Tsinghua University, Beijing, 100084 China

## Abstract

Acid-sensing ion channels (ASICs) are neuronal voltage-independent Na^+^ channels that are activated by extracellular acidification. ASICs play essential roles in a wide range of physiological processes, including sodium homeostasis, synaptic plasticity, neurodegeneration, and sensory transduction. Mambalgins, a family of three-finger toxins isolated from black mamba venom, specifically inhibit ASICs to exert strong analgesic effects in vivo, thus are thought to have potential therapeutic values against pain. However, the interaction and inhibition mechanism of mambalgin on ASICs remains elusive. Here, we report a cryo-electron microscopy (cryo-EM) structure of chicken ASIC1a (cASIC1a) in complex with mambalgin-1 toxin at 5.4 Å resolution. Our structure provides the first experimental evidence that mambalgin-1 interacts directly with the extracellular thumb domain of cASIC1a, rather than inserting into the acid-sensing pocket, as previously reported. Binding of mambalgin-1 leads to relocation of the thumb domain that could disrupt the acidic pocket of cASIC1a, illustrating an unusual inhibition mechanism of toxins on ASIC channels through an allosteric effect. These findings establish a structural basis for the toxicity of the mambalgins, and provide crucial insights for the development of new optimized inhibitors of ASICs.

## Introduction

Acid-sensing ion channels (ASICs) are proton-gated and Na^+^-selective ion channels^1–3^ that are widely expressed throughout central and peripheral nervous systems in vertebrates^4,5^ and belong to the epithelial sodium channel/degenerin (ENaC/DEG) superfamily of cation channels^6,7^. ASICs are encoded by four genes that give rise to six known isoforms (ASIC1a, ASIC1b, ASIC2a, ASIC2b, ASIC3, and ASIC4)^8^. The channels are formed by combinations of ASIC subunits in homo or hetero-trimeric complexes^9–12^, with different subunits conferring distinct properties, exhibiting a broad range of kinetic, ion selectivity and pharmacological properties^13–15^. ASICs are involved in various physiological processes, including synaptic plasticity^16,17^, neurodegeneration^15^, and pain sensation^2,8,18–20^. ASICs therefore have emerged as new potential therapeutic targets in the management of psychiatric disorders, neurodegenerative diseases and pain^2^.

ASICs are subject to modulation by intracellular pH^21^, extracellular alkalosis^22–24^, and various other factors^25^. Small modulators such as amiloride can act on ASICs as non-specific blockers^26^. Several peptide toxins have been identified as selective and potent modulators for ASICs and function as channel agonizts, such as Texas coral snake toxin MitTx^27^; desensitization state promoters, such as psalmotoxin-1 (PcTx1) from the venom of the tarantula;^28,29^ or inhibitors, such as the sea anemone toxin APETx2^30^ and mambalgins isolated from mamba venom^31^. These toxins bind to open, desensitized and closed states of the channels respectively, providing powerful tools to arrest ASICs in specific conformational states for pharmacological, biophysical, and structural studies^32,33^. In recent years, crystal structures of chicken ASIC1a (cASIC1a) in different states have been reported, including structures of apo-form cASIC1a in a desensitized state^10,34^ at low-pH, PcTx1-stabilized open and desensitized states^35,36^ and a MitTx-bound open state^37^.

Mambalgin-1, a toxin isolated from black mamba venom, is a disulfide-rich polypeptide consisting of 57 amino acids and belongs to the family of three-finger toxins^31,38^. It has been reported to be a potent, rapid and reversible inhibitor of ASIC1a or ASIC1b-containing channels in both central and peripheral neurons^31^. Experiments in mice have demonstrated the analgesic effect of mambalgin-1, which is as strong as morphine but does not involve opioid receptors, so it produces fewer adverse side effects than traditional opioid drugs, indicating high significance with therapeutic value^31^.

Mambalgin-1 can bind to and stabilize ASICs in a physiologically relevant closed-channel conformation^31^, but the underlying binding and inhibition mechanism remains elusive. Structural studies of mambalgin-1 show that the toxin has a strong positive electrostatic potential domain that may contribute to its binding to ASICs^22,23,38^. Previously, a docked structure of the cASIC1a–mambalgin-1 complex was reported^23,24^, following the crystal structure of the cASIC1a–PcTx1 complex. Mambalgin-1 was predicted to insert into the acidic pocket (also known as the acid-sensing pocket) inside the extracellular domain of the ASIC, similar to the binding of PcTx1 to ASIC1a, which was also investigated through electrophysiological analysis on wild-type and mutant mambalgin-1 or ASICs^23,24^. However, PcTx1 and mambalgin-1 belong to different super-families, with low homogeneity in both sequence and structure^26^. Furthermore, electrophysiological experiments indicated that PcTx1 and mambalgin-1 modify the affinity for protons of ASIC1a in different ways^24,29,31,39^. PcTx1 binds tightly to the open and desensitized states of ASIC1a^29^, while mambalgin-1 binds to the closed and inactivated states of the channel^31^. The different structural and pharmacological properties of mambalgin-1 and PcTx1 indicate that the two toxins must bind and modulate ASICs in distinct mechanisms.

To clearly illustrate the molecular mechanism underlying interaction and modulation of mambalgin-1 on ASICs, we set out to elucidate the structure of the chicken ASIC1a (cASIC1a) in complex with mambalgin-1 using single-particle cryo-EM. Here we report cryo-EM structure of a cASIC1a–mambalgin-1 complex at a resolution of 5.4 Å. Our structure shows that mambalgin-1 interacts directly with the thumb domain of cASIC1a but not with the acid-sensing pocket as hypothesized through docking analysis based on cASIC1a–PcTx1 crystal structures^23,24^. At the same time, mambalgin-1 binding was observed to induce an obvious conformational change in the extracellular thumb domain of cASIC1a, which might disrupt the acid-sensing process in cASIC1a. Electrophysiological analysis of the wild-type and mutant mambalgin-1 and cASIC1a clearly verified these structural results. Our combination of structural and functional data illustrate a new binding mode of mambalgin-1 to ASIC1a channel, suggest an unusual allosteric inhibition mechanism of ASICs and provide a structural basis for further development of inhibitors of ASICs.

## Results

### Structure determination of cASIC1a^ΔNC^–Mambalgin-1 complex

**Fig. 1:**
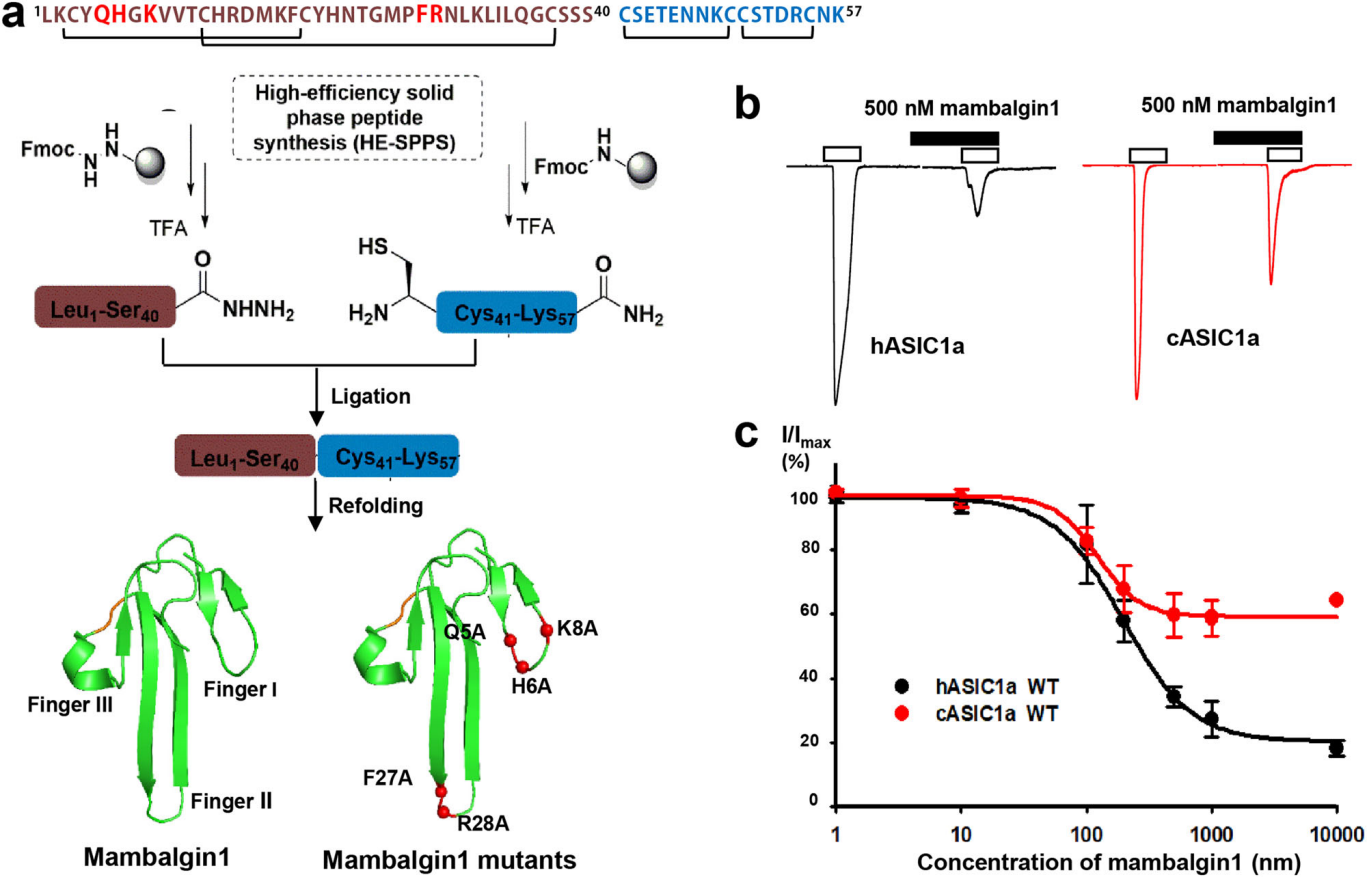
Chemical synthesis and bioactivity analysis of mambalgin-1. **a** Synthetic route of mambalgin-1 and its mutants. **b** Synthetic mambalgin-1 inhibits recombinant human and chicken ASIC1a (hASIC1a and cASIC1a) channels in CHO cells. After two stable records of acid currents that were elicited by a pH drop from 7.4 to 6.0 for 4 s, synthetic mambalgin-1 was applied at pH 7.4 for 30 s followed by another application during a 4spH drop to 6.0. **c** Concentration–response curve showing the inhibition of hASIC1a and cASIC1a channels expressed in CHO cells (*n* = 3–14) by synthetic wild-type mambalgin-1

We used an efficient, hydrazide-based native chemical ligation approach to generate large quantities (mini-gram level) of the highly pure and homogeneous mambalgin-1 toxin and its mutants^40^ (Fig. 1a and Supplementary Figure S1). Electrophysiological analysis in CHO cells showed the synthetic mambalgin-1 caused a concentration-dependent inhibition with an IC_50_ of 123.6 ± 20.3 nM for chicken ASIC1a (cASIC1a) and 197.3 ± 18.7 nM for human ASIC1a (hASIC1a) (Fig. 1b, c). These values are consistent with that reported for mambalgin-1 isolated from snake venom (hASIC1a in COS-7 cells, 127 nM)^31^. At the same time, a truncated cASIC1a (residues 14–463, denoted as cASIC1a^ΔNC^) was overexpressed and purified from sf9 cells. The binding of chemically synthesized mambalgin-1 to purified cASIC1a^ΔNC^ was analyzed using gel filtration combined with denaturing HPLC and SDS–PAGE (Supplementary Figure S2). The data clearly revealed the formation of a stable cASIC1a^ΔNC^–mambalgin-1 complex in vitro. Eventually, the highly stable and homogeneous cASIC1a^ΔNC^–mambalgin-1 complex was observed to be uniform in composition based on SDS–PAGE analysis and was therefore satisfactory for cryo-EM analysis.

**Fig. 2:**
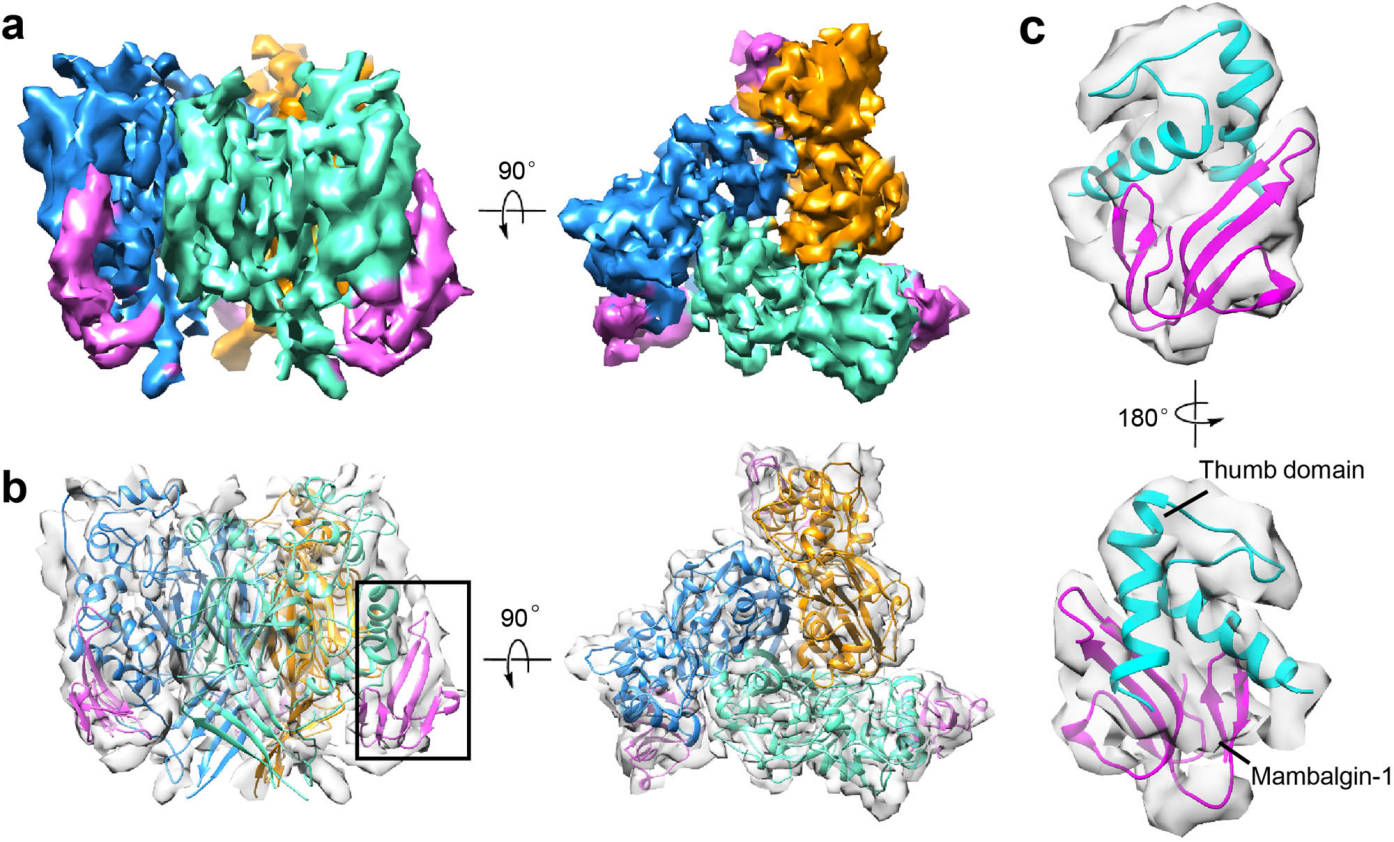
Cryo-EM structure of the cASIC1a^ΔNC^–mambalgin-1 complex. **a** Final 5.4 Å density map of the extracellular part of cASIC1a^ΔNC^-mamabalgin-1 complex in side and top view. The map of each single subunit of cASIC1a^ΔNC^ was colored in blue, cyan and yellow respectively, the maps of three mambalgin-1 molecules were colored in magenta. **b** A poly-alanine atomic model of cASIC1a^ΔNC^–mamabalgin-1 complex superimposed with the cryo-EM map displayed from two views as shown in (**a**). The model was colored by protein subunit as in (**a**). **c** The thumb domain of cASIC1a^ΔNC^ and mambalgin-1 were fitted into the cryo-EM map. The cartoon of thumb domain and mambalgin-1 were colored in yellow and magenta respectively

Single-particle cryo-EM was applied to illustrate the structure of the purified cASIC1a^ΔNC^–mambalgin-1 complex. Cryo-EM images of the complex were acquired, and homogenous particles with different orientations were collected (Supplementary Figure S3A). Through rigorous two-dimensional (2D) and three-dimensional (3D) classifications, ~100,000 particles were selected for further processing (Supplementary Figure S3B). Refinement of this subset in a fully-automated manner using RELION^41^ resulted in a cryo-EM reconstruction of the cASIC1a^ΔNC^–mambalgin-1 complex at 5.7Å resolution (Supplementary Figure S3C, S3D and S4A). The initial 3D reconstruction map showed better density at the extracellular domain of cASIC1a^ΔNC^ than at the transmembrane domain. To improve local density, the transmembrane section of cASIC1a^ΔNC^ was masked out, and local refinement of the extracellular part undertaken, eventually resulting in a density map at a resolution of 5.4 Å (Fig. 2a and Supplementary Figure S3D and S4B). Due to the relatively low local resolution, de novo structure determination of the cASIC1a^ΔNC^–mambalgin-1 complex was hindered at this point. As an alternative, we attempted to fit the density map rigidly using crystal structures of cASIC1a and mambalgin-1 as templates (Fig. 2b). Most of the secondary structure elements could be applied where there was supporting density, especially the thumb domain of cASIC1a^ΔNC^, and the finger regions of mambalgin-1 (Fig. 2b, c). However, due to the low resolution of the density map, it was inefficient to attribute the side chains of residues either of mambalgin-1 or cASIC1a molecules, thus a poly-alanine model of the cASIC1a^ΔNC^–mambalgin-1 complex was obtained eventually (Fig. 2b).

### Location of mambalgin-1 in the cASIC1a^ΔNC^–mambalgin-1 complex

**Fig. 3:**
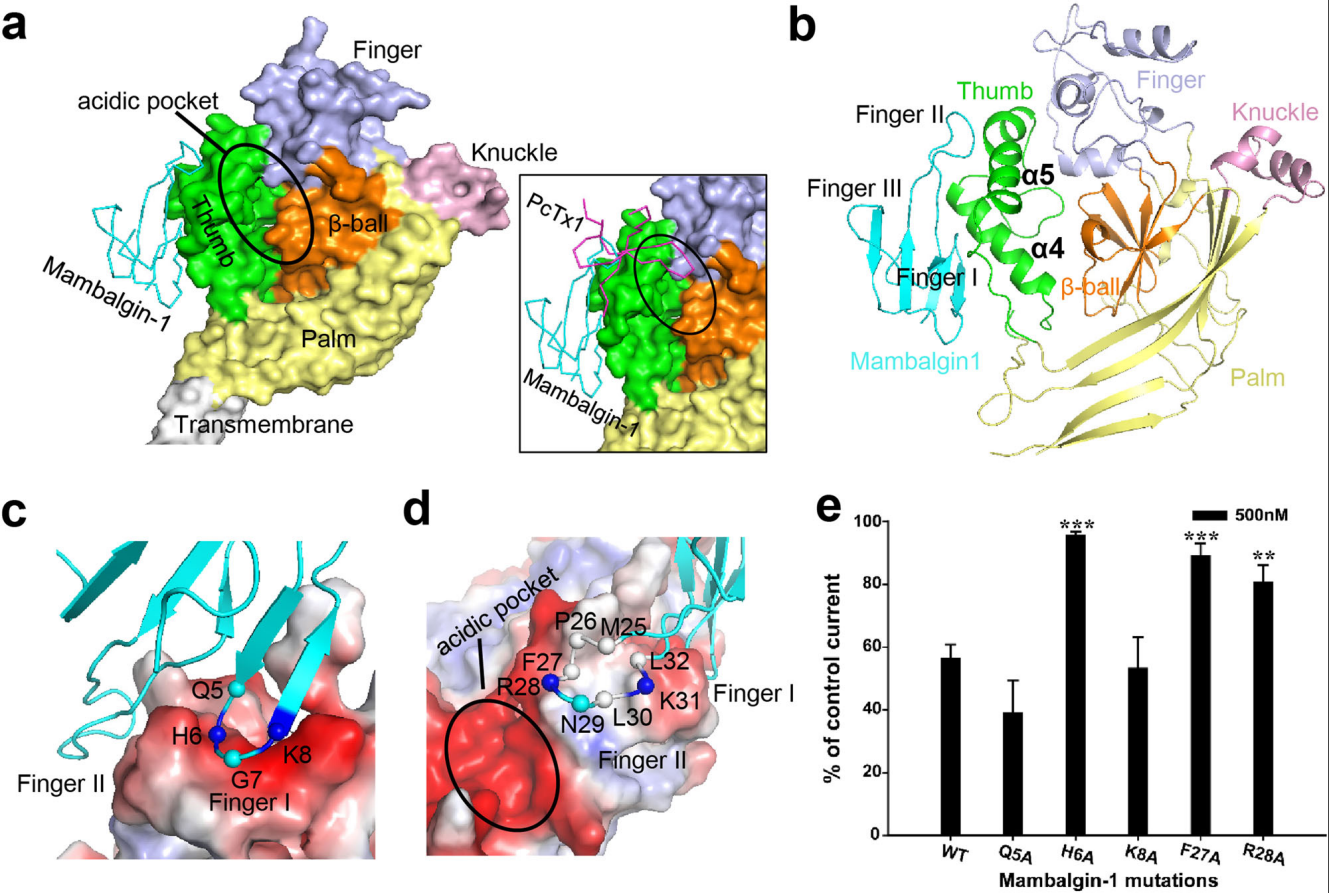
Location and interaction of mambalgin-1 on cASIC1a. **a** Location of mambalgin-1 in cASIC1a^ΔNC^-mambalgin-1 complex. A single subunit of cASIC1a channel is shown in surface representation with each domain in a different color. Mambalgin-1 is shown in ribbon representation. Insert, the location of mambalgin-1 (cyan) overlapping with PcTx1 (magenta). The PcTx1 position was taken from the structure of PcTx1-bound cASIC1a (PDB 4FZ1). **b** Cartoon representation of a single subunit derived from the cASIC1a^ΔNC^-mambalgin-1 complex. **c**, **d** Close-up views of the interactions of the Finger I region (**c**) and Finger II region (**d**) of mambalgin-1 with the thumb domain of cASIC1a^ΔNC^. The Cα atoms of several key residues in the finger regions are shown as spheres. Basic residues of mambalgin-1 fingers are colored in blue, and the hydrophobic residues in white. **e** Bar graph representing the effect of different mambalgin-1 point mutants (500 nM) on wild-type cASIC1a. Data are means±S.E. (error bars) (*P < 0.05; **P < 0.01; ***P < 0.001; different from WT; *t*-test; *n* = 4–13)

From the calculated 5.4 Å density map, detailed structures of the cASIC1a^ΔNC^–mambalgin-1 complex could be further delineated. The first observation from the cryo-EM structure of the cASIC1a^ΔNC^-mambalgin-1 complex was that mambalgin-1 binds to the thumb domain of cASIC1a directly. The overall structure of cASIC1a^ΔNC^–mambalgin-1 complex showed a triskelion-like shape when viewed down the 3-fold symmetry axis, with three mambalgin-1 molecules protruding from the edges of the channel trimer (Fig. 2b). Each mambalgin-1 molecule interacts almost exclusively with a single subunit. It was clear that mambalgin-1 only interacts with the outside surface of the thumb domain of cASIC1a, rather than inserting into the acidic pocket, or binding to the proposed interface region in the β-ball or palm domain (Fig. 3a).

The binding mode of mambalgin-1 on cASIC1a observed form our cryo-EM structure is distinct from that proposed previously^23,24^, or the binding mode of tarantula toxin PcTx1^35,36^. In previous studies, bioinformatics and mutagenesis data suggested that mambalgin-2, a homolog of mamabalgin-1, binds to cASIC1a by inserting into the acidic pocket at the interface of two subunits, principally through the upper part of the thumb domain. It was also suggested that mambalgin-2 interferes with residues of the β-ball domain at the bottom of the acidic pocket and with the upper palm domain on the adjacent subunit^24^. Our cryo-EM structure clearly showed that mambalgin-1 could only bind to the outside surface of the thumb domain of cASIC1a, but not insert into the acidic pocket or bind to the proposed interface region in the palm domain (Figs. 2b and 3a). In the other hand, the binding mode of mambalgin-1 to cASIC1a is obviously distinct from that of PcTx1 (Fig. 3a). The structure of cASIC1a in complex with PcTx1 showed that PcTx1 binds to the acidic pocket of the extracellular domain at the interface of two subunits of cASIC1a using a hydrophobic patch and a basic cluster for the interaction^35,36^. However, mambalgin-1 interacts only with the thumb domain and not with the lower palm domain or the acid-sensing pocket according to the density map, thus showing a distinctive binding interface with cASIC1a. The unexpected binding manner of mambalgin-1 to cASIC1a suggests that the inhibitory effect of mambalgin-1 on ASIC channel should not depend on the ASIC acid-sensing pocket blockage as previously proposed^23,24^.

### Interaction between mambalgin-1 and cASIC1a

From the cryo-EM structure of the cASIC1a^ΔNC^–mambalgin-1 complex, two interfaces for toxin-channel interaction were seen: the tip region of mambalgin-1 Finger I with the α4 helix of the cASIC1a thumb domain, and the tip region of Finger II with the α5 helix of the thumb domain (Fig. 3b). The Finger II-α5 interaction was previously indicated based on mutation studies on mambalgin-1 and rat ASIC1a^23^, while the Finger I-α4 interaction was previously unknown. Detailed interface analysis of cASIC1a^ΔNC^–mambalgin-1 illustrated that the finger I-α4 interaction is possibly mediated by electrostatic interactions, mainly between the basic residues His6/Lys8 in the Finger I tip region, and the acidic region of α4 helix (Fig. 3c). Meanwhile, consistent with previous studies^24^, the interaction between Finger II of mambalgin-1 and the α5 helix of the thumb domain was thought to include electrostatic interactions between the positively charged residues Arg28/Lys31 in the Finger II tip region, and the acidic region of α5 helix (Fig. 3d). A hydrophobic patch consisting of residues Met25/Pro26/Phe27/Leu30/Leu32 in Finger II could also contribute to the cASIC1a–mambalgin-1 interactions, especially with the hydrophobic region in α5 helix of cASIC1a thumb domain (Fig. 3d).

To verify the above interactions between mambalgin-1 and cASIC1a, several site mutations were introduced into both Finger I and Finger II of mambalgin-1, as well as into their possible interacting regions on the thumb domain of cASIC1a. Electrophysiological experimental data showed that mutation of mambalgin-1 in the Finger I region (Q5A, H6A, and K8A) or Finger II region (F27A and R28A) perturbed the inhibitory effects of the cASIC1a channel (Fig. 3e). On the other hand, cASIC1a mutants (R316A and Y317A, located on the α4 helix) also showed altered inhibitory responses upon mambalgin-1 binding: the Y317A mutant showed no response to the toxin binding, whereas the R316A mutant was more sensitive to mambalgin-1 than wild-type cASIC1a channel. Meanwhile, mutation of cASIC1a–Asp346Ala, Phe351Ala, and Asp356Ala (on the α5-helix in the thumb domain) was observed to reverse the toxin effect from inhibition to potentiation (Supplementary Figure S5A). pH-dependent activation of cASIC1a mutant currents were also tested. The activation of cASIC1a mutants Y317A, D346A, F351A, and D356A were shifted toward more acidic pH compared with wild-type cASIC1a, which meant that the channels had a more stable closed state (Supplementary Figure S5B). While in the presence of mambalgin-1, taking F351A mutant as a sample, the activation curve shifted toward a more alkaline pH, indicating a potentiation effect of mambalgin-1 to the F351A mutant (Supplementary Figure S5C). These data, combined with the cryo-EM structure of cASIC1a^ΔNC^–mambalgin-1 complex, exemplify the direct interaction between the Finger I/II regions of mambalgin-1 and the extracellular thumb of the ASIC1a channel, revealing an interaction mode between mambalgin-1 and ASIC channel different to that previously proposed^23,24^.

### Conformational change of the thumb domain of cASIC1a

**Fig. 4:**
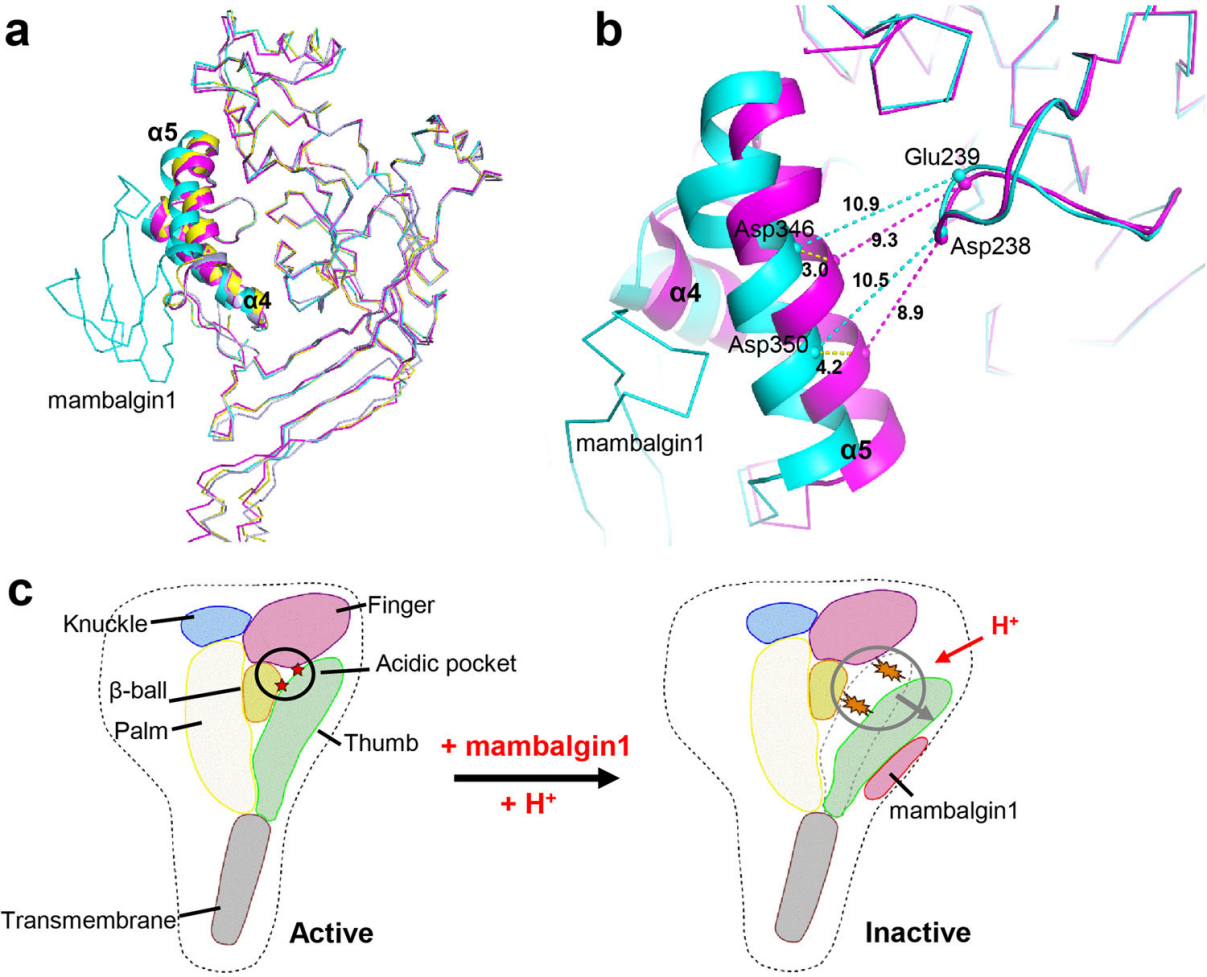
The conformational change of the thumb domain of cASIC1a. **a** Superimposition of cASIC1a in different states illustrates the movement of the thumb domain triggered by mambalgin-1 binding. The thumb domain is shown cartoon and the other parts of cASIC1a as well as mambalgin1 in ribbon. The apo-cASIC1a (PDB 4NYK), PcTx1-bound cASIC1a (PDB 4FZ1), MitTx1-bound cASIC1a (PDB 4NTY) and mambalgin-1-bound cASIC1a are colored in magenta, yellow, light blue and cyan, respectively. **b** Superimposition of the thumb domain and its adjacent regions in the β-ball domain of apo-cASIC1a (magenta) and mambalgin-1-bound cASIC1a (cyan). The Cα atoms of several key residues involved in acid sensing are shown as spheres, and the distances between these Cα atoms are indicated. **c** A proposed allosteric mechanism for the inhibition of ASIC1a channel by mambalgin-1. Mambalgin-1 binding to the ASIC1a channel triggers the movement of the thumb domain, which disrupts the connecting residue pairs in the acidic pocket and results in an expanded pocket. This conformational change may lead to the decoupling of domain rearrangement, trap the channel in a closed state. Domains of a single subunit of ASIC1a are shown as yellow for Palm, blue for Knuckle, purple for Finger, olive for β–ball, green for Thumb and gray for Transmembrane domains. The approximate position of the acidic pocket is indicated by black dashed lines. The red stars represent key residue pairs that connect β–ball and thumb domain

The cryo-EM structure of the cASIC1a^ΔNC^–mambalgin-1 complex revealed an unusual allosteric effect of mambalgin-1, wherein the thumb domain of ASIC1a functioned as an allosteric site. In comparison with the crystal structures of cASIC1a^10,35,37^, the cryo-EM structure of the cASIC1a^ΔNC^-mambalgin-1 complex showed the thumb domain of cASIC1a to undergo a significant conformation change upon mambalgin-1 binding (Fig. 4a). In the presence of mambalgin-1, relocation of the α4/α5 helixes resulted in the thumb domain of cASIC1a moving away from the channel center. Superimposition of the β-ball domain of apo-cASIC1a and mambalgin-1-bound cASIC1a showed the Cα atoms of Asp346 and Asp350, two key residues located in the α5 helix that were considered to play crucial roles in acid sensing, exhibited a shift of about 3.0 and 4.2 Å against the β-ball domain after mambalgin-1 binding (Fig. 4b). As a result, the distance between the Cα atoms of residues Asp346 and Glu239 in the cASIC1a^ΔNC^–mambalgin-1 complex was found to be longer than that in apo-cASIC1a (10.9 vs. 9.3 Å). The distance between the Cα atoms of Asp350 and Asp238 was also observed to be longer (10.5 vs. 8.5 Å) (Fig. 4b). Notably, the expansion of the thumb domain of the ASIC channel was not observed in other ASIC–toxin complex structures, such as cASIC–PcTx1 and cASIC1a–MitTx^35–37^. Crystal structures showed that binding by both PcTx1 and MitTx could trigger a conformation change of the extracellular vestibule in the ASIC channel, and stabilization of the open channel pore. The lower palm domain (but not the thumb domain) acted as a conformationally flexible, proton-sensitive domain at the core of ion channel gating. Considering that mambalgin-1, PcTx1 and MitTx belong to different toxin families, the allosteric inhibition of cASIC1a by mambalgin-1 represents a novel modulation mode of ASIC channels by peptide toxins.

## Discussion

The cryo-EM structure of cASIC1a^ΔNC^–mambalgin-1 complex, especially the extracellular domain of cASIC1a^ΔNC^, recapitulated all hallmark features of the extracellular domain of cASIC1a in previously reported crystal structures^10,34–37^. For the interaction between the inhibitory toxin mambalgin-1 and ASIC channel, the cryo-EM structure provided us with new observations distinct with previously proposed. The first observation from the structure was that mambalgin-1 binds to the thumb domain of cASIC1a directly (Fig. 3a). The cASIC1a^ΔNC^–mambalgin-1 complex structure reported here unambiguously showed that mambalgin-1 only interacts with the outside surface of the thumb domain of cASIC1a, rather than inserting into the acidic pocket, or binding to the proposed interface region in the β-ball or palm domain (Fig. 3a). This observation is distinct from the proposed binding mode of mambalgin-1 on ASIC^23,24^, or the binding mode of PcTx1^35,36^. Furthermore, the cASIC1a^ΔNC^–mambalgin-1 complex structure revealed that, in addition to Finger II, the Finger I of mamalgin-1 could act as an important hot spot for the channel-toxin interaction, which was previously unknown. A detailed interface analysis showed that Finger I of mambalgin-1 could interact with the α4 helix of the cASIC1a thumb domain (Fig. 3c). Electrophysiological experimental data showed that mutation of mambalgin-1 in the Finger I region (Q5A, H6A, and K8A) perturbed the inhibitory effects of the cASIC1a channel. It is possibly that the Finger I/α4 interaction is mediated by electrostatic interactions, mainly between the basic residue His6 in the Finger I tip region, and the acidic region of α4 helix (Fig. 3e). On the other hand, cASIC1a mutants (R316A and Y317A, located on the α4 helix) also showed altered inhibitory responses upon mambalgin-1 binding: the Y317A mutant showed no response to the toxin binding, whereas the R316A mutant was more sensitive to mambalgin-1 than wild-type cASIC1a channel (Supplementary Figure S5A). Altogether, these data are additional evidence for the Finger I/α4 interaction. Meanwhile, consistent with previous studies^23^, the interaction between Finger II of mambalgin-1 and the α5 helix of the thumb domain was also observed in our structure. These are thought to include electrostatic interactions between the positively charged residues Arg28/Lys31 in the Finger II tip region, and the acidic region of α5 helix. A hydrophobic patch consisting of residues Met25/Pro26/Phe27/Leu30/Leu32 in Finger II could also contribute to the mambalgin-1–cASIC1a interactions, especially with the hydrophobic region in α5 helix of cASIC1a thumb domain (Fig. 3d). These data, combined with the cryo-EM structure of cASIC1a^ΔNC^–mambalgin-1 complex, exemplified the direct interaction between the Finger I/II regions of mambalgin-1 and the extracellular thumb of the ASIC1a channel, revealed an interaction mode between mambalgin-1 and ASIC channel different to that previously proposed^23,24^. The unexpected binding manner of mambalgin-1 to cASIC1a suggests that the inhibitory effect of mambalgin-1 on ASIC channel should not depend on the ASIC acidic pocket blockage.

Moreover, an unusual conformation change in the extracellular thumb domain of cASIC1a was clearly observed in the cASIC1a^ΔNC^–mambalgin-1 complex structure. This observation provided us clues to unveil the mechanism for the inhibitory effect of mambalgin-1 on cASIC1a channel. Mambalgin-1 binding to the thumb domain induced the α4/α5 helixes of the thumb domain moving away from the ASIC1a channel center, resulting in an expansion of the acidic pocket, which was enclosed by the thumb, the β-ball and the finger domains. We propose that the conformation change providing the structural basis for the inhibition of ASIC channel by toxin. It was reported that, in the acidic pocket, acidic residues allow the fine tuning of ASIC pH dependence^42^. The acidic pocket underwent conformational changes during both activation and desensitization of ASIC channel^42^. Voltage clamp fluorometry (VCF) analysis provided evidence of conformational changes in the acidic pocket correlated to activation and desensitization, and proposed a likely sequence of conformational changes in the acidic pocket upon extracellular acidification^42^. Furthermore, studies with ASIC1a–ASIC2a chimeras showed that swapping the thumb domain between subunits results in faster channel desensitization^43^. Likewise, the covalent modification of Cys residues at selected positions in the β-ball–thumb interface accelerated the desensitization of the mutant channels^43^. A recent luminescence resonance energy transfer (LRET) study^44^ and studies of accessibility with thiol-reactive reagents revealed that the β-ball and thumb domains reside apart in the resting state but that they become closer to each other in response to extracellular acidification^43^. It was proposed that the thumb domain moves upon continuous exposure to an acidic extracellular milieu, assisting with the closing of the pore during channel desensitization^43^.

Our structure exemplified the direct interaction between the thumb domain of cASIC1a and mambalgin-1, and revealed an unusual conformation change of the thumb domain. Upon mambalgin-1 binding, the thumb domain moved far away from the β-ball and the finger domains, disrupting the interaction between the thumb and finger/β-ball domains, thus destroyed the acidic pocket of ASIC channel (Fig. 4c). It was reasonable that ASIC with a collapse acidic pocket could have no response to extracellular acidification, resulting in the inactivation of the channel. Taken together, we conclude that peptide toxin mambalgin-1 inhibits acid-sensing ion channels through an unusual allosteric effect, taking the extracellular thumb domain as an allosteric site. This conformational change of the thumb domain led to the decoupling of subdomain rearrangement of the extracellular region of ASICs, thus trapping the channel in a closed state. In the context of this hypothesis, we suggest that the level of conformational change in the thumb domain of ASIC1a channel upon mamablgin-1 binding could determine the strength of the inhibitory effect of the toxin on ASICs from different species. The observation that human ASIC1a channel was inhibited much more complete than the chicken channel by mamablgin-1 (Fig. 1b, c) may indicate that mambalgin-1 could induce a more pronounced and stable conformational change in human ASIC1a than in chicken ASIC1a.

In summary, on the basis of chemical synthesis of peptide toxin mambalgin-1, we reported herein for the first time the structure of cASIC1a–mambalgin-1 complex determined using single-particle cryo-EM. The structure of the cASIC1a–mambalgin-1 complex revealed, surprisingly, that mambalgin-1 binds to the outer face of the extracellular thumb domain of cASIC1a directly, rather than inserting into the acidic pocket. Both Fingers I and II of mambalgin-1 contribute to the interaction between mambalgin-1 and the cASIC1a channel. Finally, upon mambalgin-1 binding, the extracellular thumb domain of cASIC1a undergoes a conformation change that may lead to the decoupling of subdomains in the extracellular region of ASICs, especially the thumb-finger-β-ball domains, thus trapping the channel in a closed state. These findings definitively establish the mechanism of the ASIC channel inhibition by mambalgins to be allosteric for the first time, and should inform the development of mambalgin-1 toxin-based analgesic agents for the treatment of neuropathic pain, without the devastating side-effects associated with opioids.

## Materials and methods

### Chemical synthesis of mambalgin1 and its mutants

Mambalgin-1 and its Alanine variants containing 57 amino acids were divided into two segments, namely [Leu1-Ser40]-NHNH2 and [Cys41-Lys57]-NH2, as shown in the synthetic route (Fig. 1b). All the peptide segments were performed using standard Fmoc SPPS protocols under microwave conditions (CEM Liberty Blue) at a 0.25 mmole scale, using a four-fold excess of Fmoc-amino acid relative to the preloaded Fmoc-NHNH2 resin and Rink Amide AM resin (0.5 mmol/g). The segments of Mambalgin-1 were ligated using hydrazide based native chemical ligation^45–47^. The full-length Mambalgin-1 peptide was directly dissolved in deionized water at 10 μM, then 100 μM GSSG, 1000 μM GSH were added sequentially and the pH was adjusted to 7.8, finally the reaction buffer was incubated at 25 °C for 12 h. The folded mambalgin-1 and its Alanine variants were characterized with reversed-phase high-performance liquid chromatography (RP-HPLC) and electrospray ionization mass spectrometry (ESI-MS), as shown in Supplementary Figure S1.

### Protein expression and purification

The truncated cASIC1a protein (residues 14-463, denoted as cASIC1a^ΔNC^) was expressed and purified, as previously described^35^. Briefly, The construct cASIC1a^Δ^^N^^C^ was derived from the chicken ASIC1 gene and was expressed as an N-terminal fusion with octa-histidine-tagged enhanced green fluorescent protein (EGFP) using baculovirus expression systems in insect cells with a TEV site, encoded upstream of cASIC1a^ΔNC^, generating a final protein sequence containing residues Gly 14 to Arg 463. Sf9 cells expressing cASIC1a^ΔNC^ protein were collected by centrifugation (3000 × *g*) and sonicated in the presence of 150 mM NaCl, 20 mM Tris (pH 7.5) and protease inhibitors, then subsequently solubilized in 2% (w/v) n-dodecyl-b-D-maltoside (DDM) for 1 h at 4 °C. The solubilized material was clarified by centrifugation (20,000 × *g*) for 1 h at 4 °C and the supernatant was incubated with Ni-NTA resin for 1 h at 4 °C. Bound protein was then eluted with 150 mM NaCl, 20 mM Tris (pH 7.5), 500 mM imidazole and 0.05% (w/v) DDM. Cleavage of the histidine-tagged GFP was achieved by TEV. The resulting cASIC1a^ΔNC^ protein was further separated using a Superdex 200 column in buffer containing 150 mM NaCl, 20 mM Tris–HCl (pH 7.5), 0.02% (w/v) DDM. The peak corresponding to trimeric cASIC1a protein was collected and transferred to the amphipols, as previously described^48^. To construct cASIC1a–mambalgin-1 complex, the cASIC1a^ΔNC^ protein and mambalgin-1 was mix in a molar ratio of 1:1.5 and incubated for 1 h, and then purified using a Superdex 200 column in running buffer composed of 150 mM NaCl, 20 mM Tris–HCl (pH 7.5).

### Cryo-EM sample preparation and data collection

Purified cASIC1a^ΔNC^–mambalgin-1 complex was concentrated to 0.6 mg/ml. After a short centrifugation at 12,000 × *g* for 10 min, we applied a droplet of 4 μl protein to H_2_/O_2_ plasma-cleaned quantifoil R2/1 copper 300 mesh grids (Quantifoil, Micro Tools, Germany). The grids were blotted for 4–6 s at 22 °C and flash-frozen in liquid ethane with an FEI vitrobot. The cryo sample could then be stored in liquid nitrogen for further screening or data collection. All the cryo-EM data were collected on an FEI Titan Krios microscope at 300 kV with a K2 camera. Micrographs were recorded at a nominal magnification of 22,500 × and a pixel size of 0.66 Å in super-resolution mode, with the defocus ranging from −1.5 μm to −3.5 μm. With a dose rate of ~6.9 e^−^/Å^2^ per second, each frame was exposed for 0.25 s. After a total exposure time of 8 s, each movie contained 32 frames, and the total dose was ~55 e^−^/Å^2^.

### Image processing

We collected 2000 micrographs, and all the micrographs were two-binned and motion corrected with MotionCorr software^49^. CTF parameter estimation was carried out with CTFFIND3^50^. Approximately 500 K particles were semi-autopicked with e2boxer software in swarm mode^51^. To increase the low-frequency signal of the particles for alignment, all the particles were further binned to a pixel size of 2.64 Å during particle extraction. After a few rounds of 2D classification to remove ice contamination and bad particles, the 231 K remaining particles were then subjected to further 3D classification with an artificial triangular prism as the initial model. Among the four classes, one class contained most of the particles (134 K) and showed great similarity to reported cASIC1a crystal structures^10,34–37^, especially the outer membrane part. This class was subjected to a further round of 3D classification, and two of the 4 classes were selected for further processing. After re-centering the particles, the selected dataset was then subjected to 3D refinement with the imposition of C3 symmetry. As the density of the transmembrane domain was quite poor, we created a soft mask focusing on the extracellular part for further refinement and got a final 3D reconstruction. The final 3D reconstruction produced a resolution of 5.7 Å for the overall map and 5.4 Å for the soft-masked map. All 2D and 3D classifications and refinements mentioned above were performed with Relion1.4^41^. All resolutions were estimated by the gold-standard Fourier shell correlation (FSC) = 0.143 criterion^52^. ResMap^53^ was used to calculate the local resolution map.

### Model building

The structural modeling for the cASIC1a^ΔNC^–mambalgin-1 complex comprised rigid-body fitting of the cASIC1a extracellular domain and full-length mambalgin-1 extracted from the crystal structures (PDB code: 4FZ1 and 5DU1) into the cryo-EM density, followed by fitting of the thumb domain (residue numbers: 305–323 and 337–359) of cASIC1a in rigid-body refinement against the density map by using COOT^54^. Furthermore, docked as a rigid-body model, Finger II (residue numbers: 23–34) of mambalgin-1 was refined in real space by using COOT, including locating the loop region into density. All the figures were created by UCSF Chimera^55^ and PyMol.

### Plasmid construction, cell culture and transient transfection of CHO cells

The coding sequence for wild-type cASIC1a was sub-cloned into the pcDNA3.1/Zeo( + ) vector. All site-directed mutations were generated with overlap PCR and inserted into pcDNA3.1/Zeo(+). The mutants were sequenced to verify that no unwanted mutations had been introduced. Chinese hamster ovary (CHO) cells were cultured in DMEM/F12 medium (Gibco) supplemented with 10% fetal bovine serum (FBS), 100 U/mL penicillin, and 100 U/mL streptomycin at 37 °C in a 5% CO_2_ incubator. The CHO cells were transferred to 24-well plates for transfection. When the CHO cells reached 90% confluence, they were transfected with 0.6 μg of plasmid encoding EGFP and 0.8 μg of plasmid encoding wild-type or mutant cASIC1a using Lipofectamine 2000 (Invitrogen, USA). After incubation for 5 h, the cells were transferred to poly-L-lysine (Sigma)-coated slides for culture for another 24–48 h in fresh medium. They were then used for the electrophysiological analysis.

### Electrophysiological analysis of CHO cells

For the whole-cell recordings, the bath solution contained 150 mM NaCl, 4 mM KCl, 2 mM CaCl_2_, 1 mM MgCl_2_, and 10 mM HEPES (pH 7.4, ~308 mOsm). For acidic solution below pH 6.0, HEPES was replaced by 10 mM MES to buffer solutions. The electrodes were pulled from thick-walled borosilicate glass capillaries with filaments (1.5 mm diameter; Sutter Instruments) on a four-stage puller (P-1000; Sutter, USA) and had resistances of 2–5 MΩ when filled with intracellular solution containing 140 mM KCl, 10 mM NaCl, 5 mM EGTA, 10 mM HEPES, (pH 7.4, ~297 mOsm). All chemicals were obtained from Sigma. The experiments were performed at room temperature with an EPC-10 amplifier (HEKA Electronic) with the data acquisition software PatchMaster. Membrane potential was held at −70 mV in all experiments. Acid-induced currents were recorded by rapidly exchanging local solution from pH 7.4 to acidic pH through a Y-tube perfusion system. Toxins were applied 30 s before the pH decreased and persisted during low pH application. Channels were activated by acid perfusion at least every 2 min to allow for a complete recovery of the channels from desensitization. Recordings in which access resistance or capacitance changed by 10% during the experiment were excluded from data analysis. Mambalgin-1 was added when the currents were stable.

### Patch-clamp electrophysiological data analysis

The data were analyzed with Clampfit and SigmaPlot. The dose–response curves used to determine the IC_50_ values were fitted using the Hill equation: *y* = 1 + (*I*_max_−1)/(1 + (IC_50_/*x*)^*h*^), where *y* represents the normalized current, Imax represents the control current of ASIC1 detected in each set of experiments; *x* is the toxin concentration, *h* is the Hill coefficient, and IC_50_ is the half-maximal concentration. The results are presented as the means ± standard errors (SE), and *n* is the number of experiments.

### Accession codes

The three-dimensional cryo-EM density map has been deposited in the Electron Microscopy Data Bank under the accession number EMD-6900 for the 5.4 Å map, and EMD-7296 for the 5.7 Å map.

## Electronic supplementary material

Supplementary Information
