# Supplementary material for: Cryo-EM structure of the ASIC1a–mambalgin-1 complex reveals that the peptide toxin mambalgin-1 inhibits acid-sensing ion channels through an unusual allosteric effect
Source: Cell Discov. 2018 Jun 5;4:27. doi: 10.1038/s41421-018-0026-1 (PMC5986765; doi:10.1038/s41421-018-0026-1)
Supplement: Supplementary file 1 — Supplementary Information [file 41421_2018_26_MOESM1_ESM.docx]

**Cryo-****EM Structure of the ASIC1a-Mambalgin-1 Complex Reveals that the** **Peptide Toxin Mambalgin-1 Inhibits Acid-Sensing Ion Channels through an Unusual Allosteric Effect**

Demeng Sun^1, 4^, You Yu^2, 4^, Xiaobin Xue^2, 4^, Man Pan^3, 4^, Ming Wen^1^, Xiaorun Li^1^, Qian Qu^3^, Siyu Li^1^, Longhua Zhang^1^, Xueming Li^2^, Lei Liu^3*^, Maojun Yang^2*^, Changlin Tian^1^^*^

*^1^School of Life Sciences, University of Science and Technology of China, Hefei 230026, China；^2^Ministry of Education Key Laboratory of Protein Science, Tsinghua-Peking Joint Center for Life Sciences, Beijing Advanced Innovation Center for Structural Biology, School of Life Sciences, Tsinghua University, Beijing 100084, China；^3^Ministry of Education Key Laboratory of Bioorganic Phosphorus Chemistry and Chemical Biology, Department of Chemistry, Tsinghua University, Beijing 100084, China.*

^4^These authors contribute equally to this work.

^*^Correspondence: Changlin Tian

Tel: +86-551-63600872;

E-mail: cltian@ustc.edu.cn

or Maojun Yang

Tel: +86-10-62789400

Email: maojunyang@ tsinghua.edu.cn

or Lei Liu

Tel: +86-10-62780027

Email: [lliu@mail.tsinghua.edu.cn](mailto:lliu@mail.tsinghua.edu.cn)

Running Title: EM Structure of ASIC1a-Mambalgin-1 Complex

**Supplementary information**


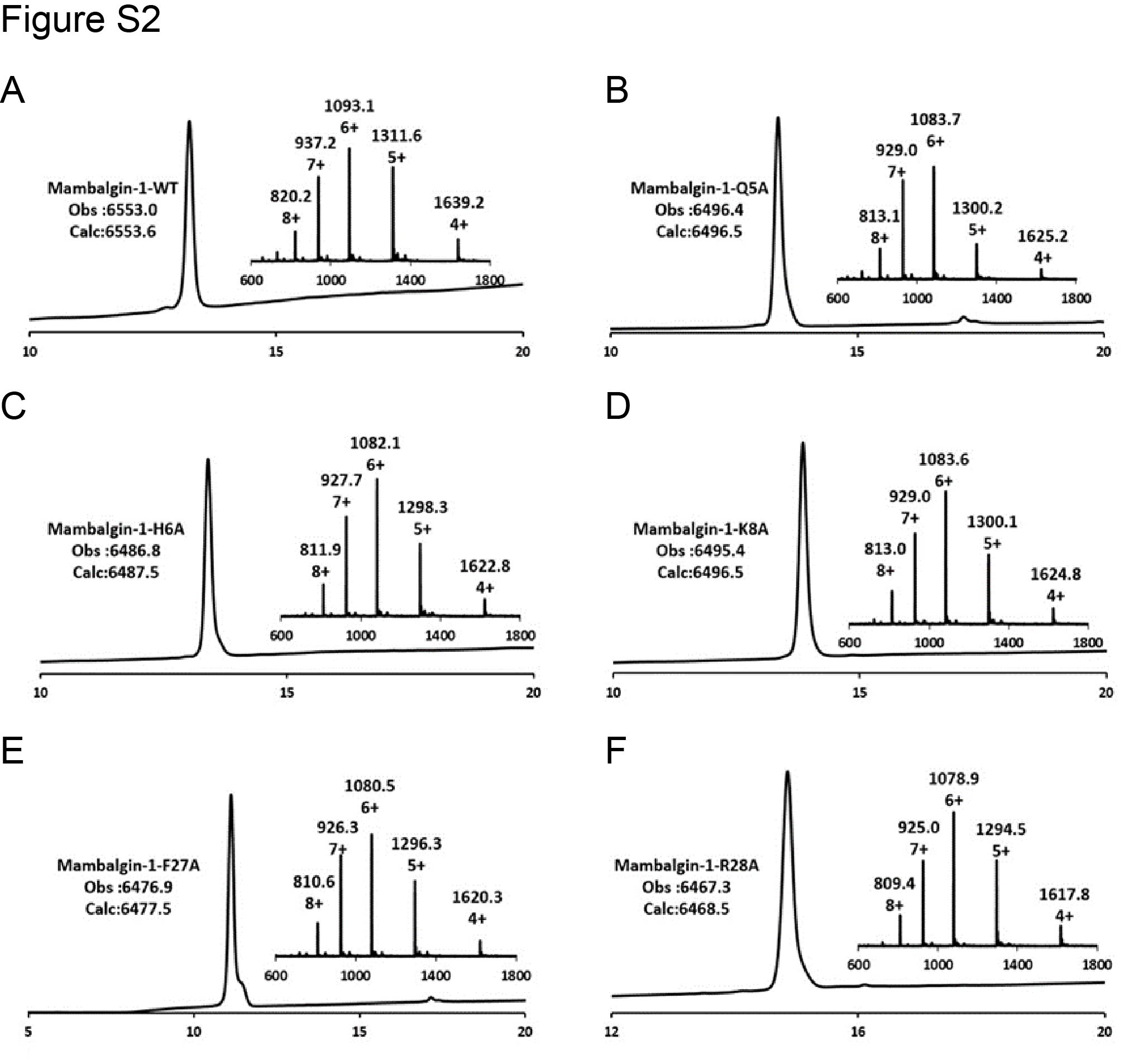


**Figure S1** Analytical HPLC chromatogram (λ = 214 nm) and observed ESI-MS of the main peak of isolated Mambalgin-1 and its mutants.


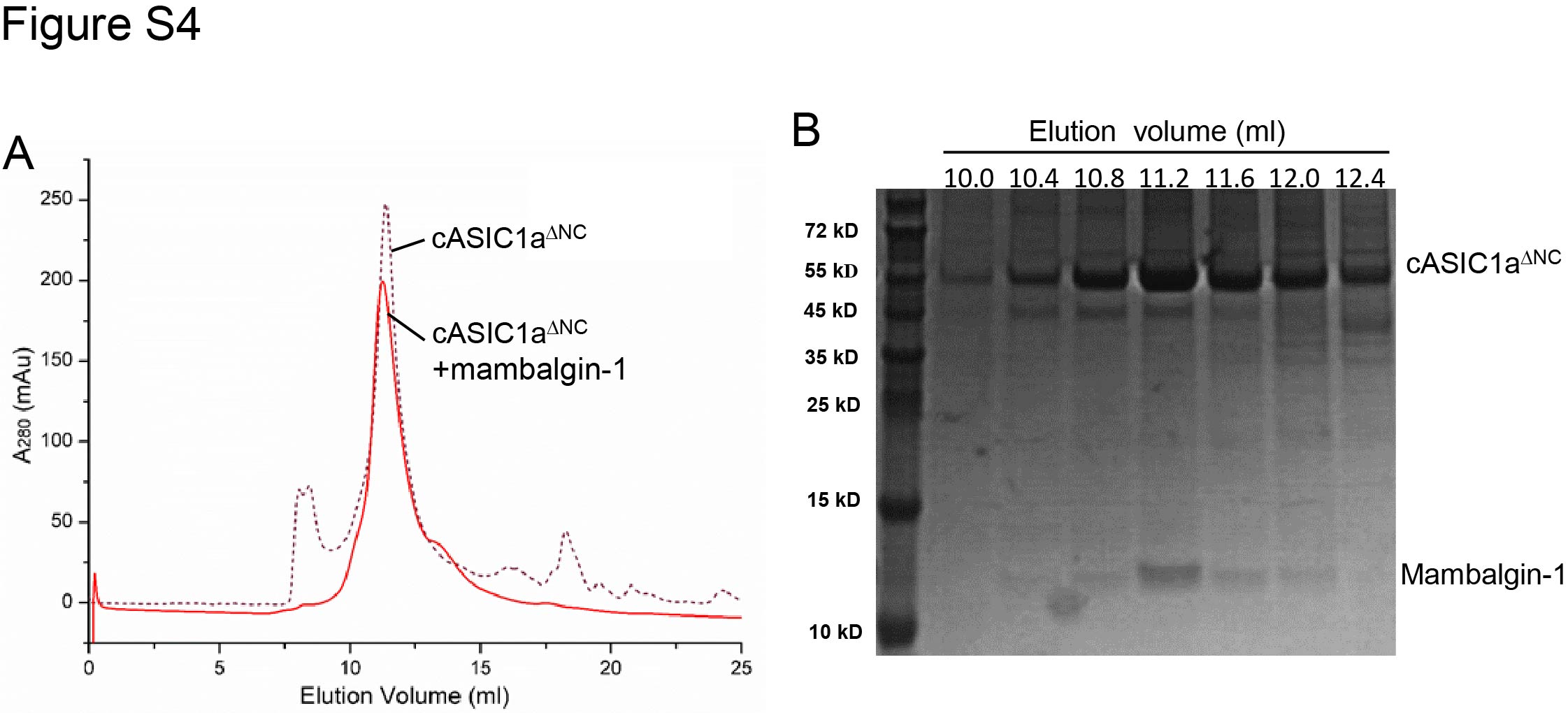


**Figure** **S2** Purification of the cASIC1a^ΔNC^-mamabalgin-1 complex. **(A)** Gel-filtration profile of apo-cASIC1a^ΔNC^ (dashed line) and the cASIC1a^ΔNC^-mamabalgin-1 complex (red line). **(B)** SDS–polyacrylamide gel electrophoresis of the peak fraction of cASIC1a^ΔNC^-mamabalgin-1 complex stained with Coomassie blue, the elution volume of each sample was labeled.


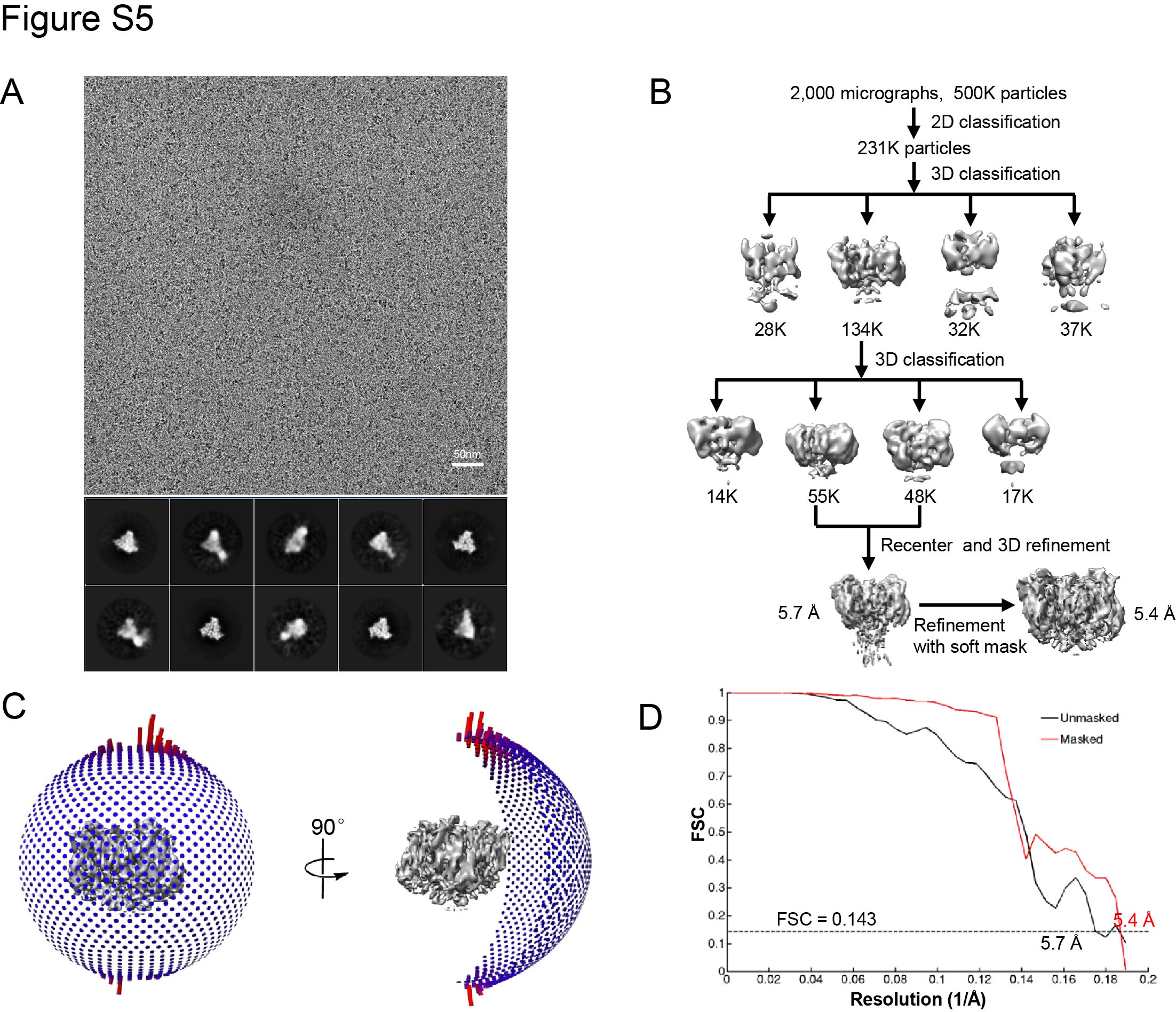


**Figure** **S3** Cryo-EM structure determination of cASIC1a^ΔNC^-mamabalgin-1 complex. **(A)** A typical micrograph of the cASIC1a^ΔNC^-mamabalgin-1 complex after drift correction. Also shown are representative 2-dimensional class averages of the particles. **(B)** Workflow of 3D classification and refinement of cryo-EM particles. A total of ~231,000 particles were kept after 2D classification, and subject to two rounds of 3D classification. A final data set containing ~ 100,000 particles were used for high-resolution refinement (see Methods for more details). **(C)** Euler angular distribution of all the particles included in the final 3D reconstruction. Red cylinders mean more particles on these orientations. Heights of cylinders represent the relative numbers of particles. **(D)** “Gold- standard” Fourier shell correlation (FSC) curves of unmasked (black) and masked (red) maps calculated from two independent halves of data sets.


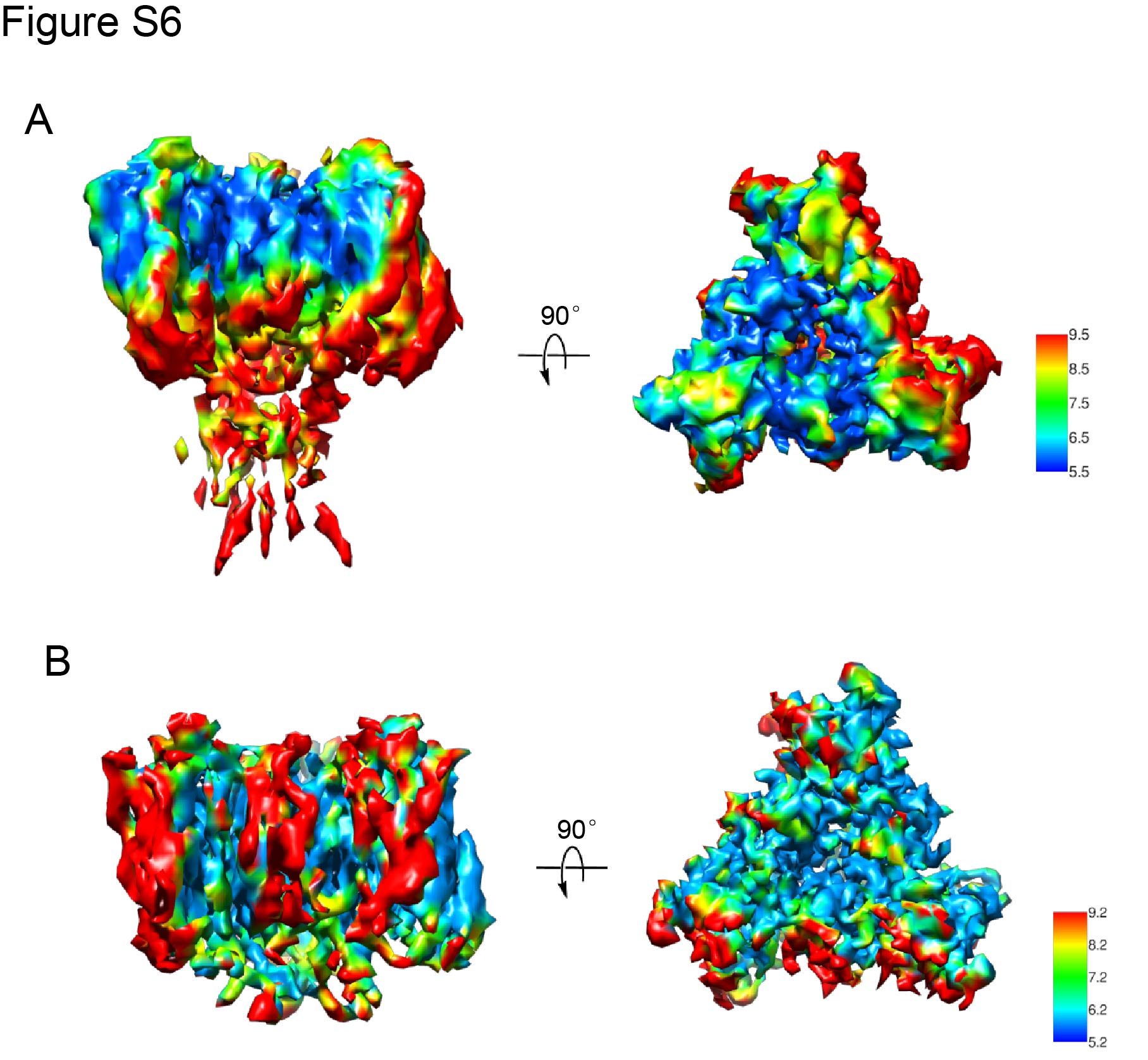


**Figure S4** Local resolution map of the final 3D density map**.** Local resolution of the unmasked (A) and masked (B) map estimated by RESMAP**.**


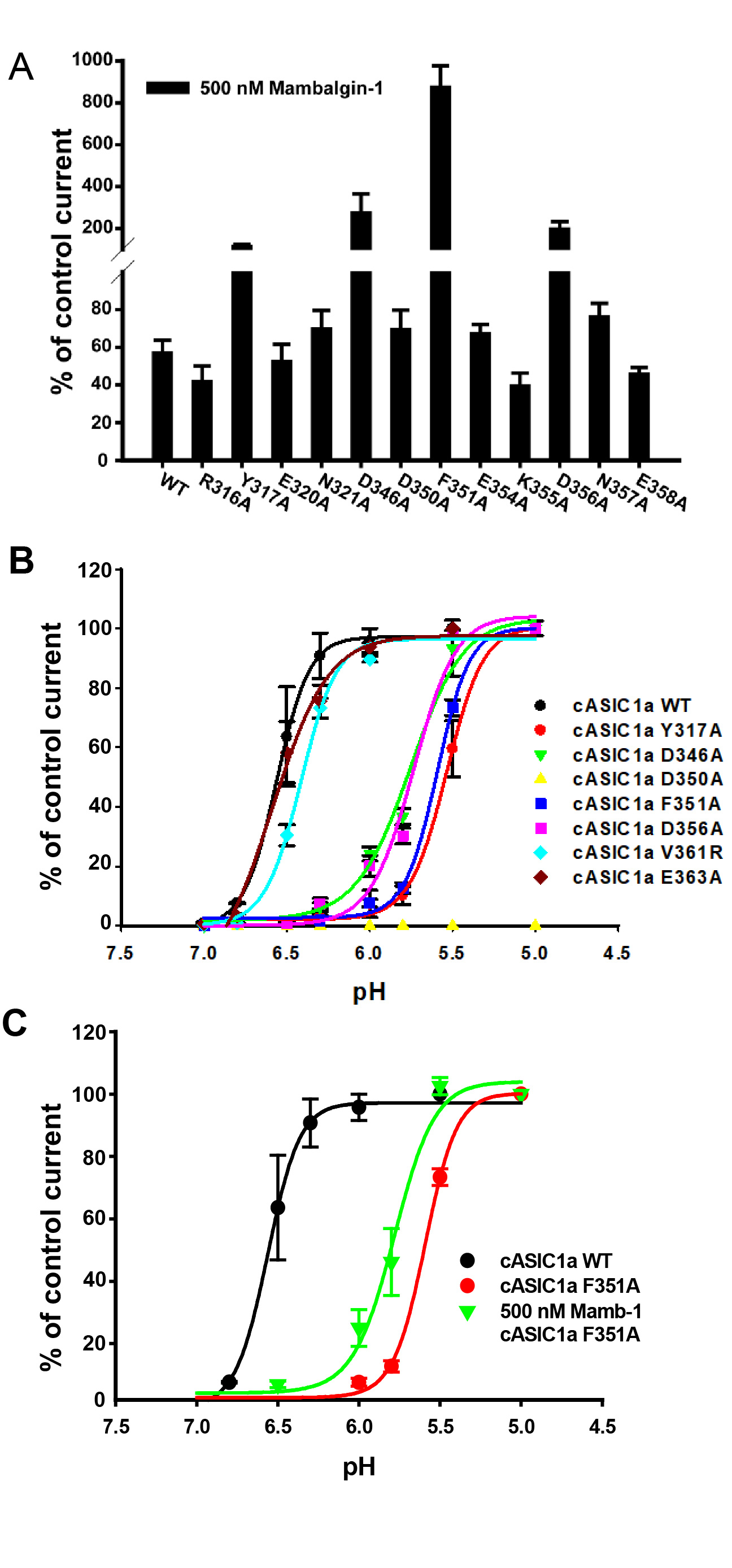


******

*******

*******

*******

*******

**Figure S5 （A）**Bar graph representing the effect of mambalgin-1 (500 nM) on different cASIC1a point mutants. Data are means ±S.E. (error bars) (*P < 0.05; *P < 0.01; ***P < 0.001; different from WT; t-test; n=3-8). **（B）**pH-dependent activation of cASIC1a mutant currents. Solid lines are fits of the mean values of each data point to a sigmoidal dose-response curve with variable slope. **(C)** pH-dependent activation of cASIC1a F351A mutant currents with (green line) and without mambalgin1(red line).
